# Changes in Perceived Accessibility to Healthcare from the Elderly between 2005 and 2014 in China: An Oaxaca–Blinder Decomposition Analysis

**DOI:** 10.3390/ijerph16203824

**Published:** 2019-10-10

**Authors:** Tao Zhang, Jing Liu, Chaojie Liu

**Affiliations:** 1 Department of Health Management, School of Medicine and Health Management, Tongji Medical College, Huazhong University of Science and Technology, Wuhan 43003, China; d201881350@hust.edu.cn; 2 Department of Health Information Management, School of Medicine and Health Management, Tongji Medical College, Huazhong University of Science and Technology, Wuhan 43003, China; 3 School of Psychology and Public Health, La Trobe University, Melbourne 3086, Australia; C.Liu@latrobe.edu.au

**Keywords:** access to healthcare, elderly, Oaxaca–Blinder decomposition, China

## Abstract

Elderly people are characterized with high needs for healthcare, accompanied by high barriers in access to healthcare. This study aimed to identify temporal changes in access to healthcare and determinants of such changes from the elderly in China, over the period between 2005 and 2014. Two waves (2005 and 2014) of data were extracted from the Chinese Longitudinal Healthy Longevity Survey (CLHLS), measuring changes in perceived accessibility to healthcare when needed by the elderly (≥65 years). The effects of the explanatory variables (need, predisposing and enabling factors) on the changes were divided into two components using the Oaxaca–Blinder decomposition method: (1) the endowment portion as a result of distribution differences of the explanatory variables and (2) the coefficient portion as a result of differential responses of the dependent variable to the explanatory variables. Perceived accessibility to healthcare from the elderly increased from 89.6% in 2005 to 96.7% in 2014. The coefficient portion (82%) contributed more to the change than the endowment portion (63%) after adjustments for a negative interaction effect (−45%) between the two. Lower perceived accessibility was associated with older age, lower income, lower affordability of daily expenses and lower insurance coverage. But the coefficient effects suggested that their impacts on perceived accessibility to healthcare declined over time. By contrast, the impacts of gender and out-of-pocket payment ratio for medical care on perceived accessibility to healthcare increased over time. Perceived accessibility to healthcare from the elderly improved between 2005 and 2014. Gender gaps are closing. But the increased effect of out-of-pocket medical payments on perceived accessibility to healthcare deserves further investigation and policy interventions.

## 1. Introduction

Equal access to healthcare services for those in need is a fundamental target of the universal health care campaign [1,2]. The elderly populations usually have a higher need for healthcare due to the aging process and deteriorated health. They also tend to have higher prevalence of ill health conditions, including multiple non-communicable diseases (NCDs) and disabilities, compared with the general population [3,4]. Over the past few decades, China has witnessed a rapid aging process: 11.4% of its population reached 65 years or older in 2017 [5]. The size of the elderly population (≥65 years) is expected to reach 329 million in 2050, accounting for 29% of the entire population in China [6]. NCDs were reported by approximately 54% of elderly people (≥65 years) in 2017, becoming a leading cause of death and loss in disability-adjusted life years (DALYs) [7]. The World Bank predicted that an aging population may further increase the burden of NCDs by 40% in China by 2030 [7].

Unfortunately, healthcare accessibility of elderly people is often short of expectations. It is common for the elderly to struggle with financial affordability of healthcare due to loss of work capabilities and income [8,9]. Meanwhile, many of them may also face further barriers in access to healthcare as a consequence of cognitive impairments and declined mobility [10,11]. The China National Health Services Survey shows that the elderly people in China are more likely to abandon the medical care they need, in comparison to younger populations [12]. About 16.2% of the elderly were found in a 2009 study to have ignored hospital admission advices from their doctors [13]. Therefore, monitoring and improving healthcare accessibility of the elderly has become a major public health function in China [12].

The Chinese government has been praised for its great efforts in developing social health insurance since 2003, as an instrument to improve accessibility of healthcare for its citizens. Within the first decade, health insurance programs had covered almost the entire population in mainland China. This was supplemented by a medical assistance scheme for catastrophic health expenditure [14] and a new rural pension scheme (NRPS), disproportionally benefiting the elderly, especially those living with a low income [15]. Empirical evidence shows that the health insurance programs increased the use of healthcare services by the elderly [16]. In rural areas, the NRPS brought in further incentives for the elderly to use healthcare services [17]. However, the development of these programs depends heavily on the financial capacity of the local governments, leading to persistent socioeconomic inequalities in healthcare services across regions. The elderly residing in low socioeconomic regions and rural areas are more likely to forfeit healthcare services when needed than their wealthy counterparts [6,12].

Extensive studies have been conducted to explore determinants of the use of healthcare services in China [10,18]. For example, Zhang and his colleagues [18] recruited 13,043 migrants aged 60 years and older from 348 cities and 10,300 communities by adopting a multi-stage stratified probability proportionate to size sampling strategy. They found household income, size of friend network, health insurance type and chronic disease status were significantly associated with health services utilization. But it remains unclear how these determinants contribute to changes, if any, in the use of healthcare services. Social health insurance programs, for example, can help release the unmet healthcare needs of the consumers [14]. But when a universal coverage is achieved, variations in health services can hardly be explained by the insurance programs. Policy makers need to identify additional priority areas for improving equity in healthcare services.

This study aimed to identify the temporal change in access to healthcare by the elderly over a ten-year period (2005–2014) in China, as well as the changing effects of its determinants over time using the Oaxaca–Blinder decomposition approach. Findings of the study will shed some light on future priorities for further improvements in healthcare accessibility.

## 2. Methods

### 2.1. Study Participants and Data Source

Data were extracted from the Chinese Longitudinal Healthy Longevity Survey (CLHLS), which monitors the health status of the elderly (≥65 years) people in China and its social, behavioral, and biological determinants [19]. Since 1998, the CLHLS has collected seven waves of data. Each wave of survey has involved new participants, in addition to those who completed the previous survey [20]. The questionnaire for CLHLS includes a large number of variables, such as basic information, health status, family status, life style, health care services and so on. We were granted ethical approval for our study: The use of CLHLS data was approved by the Biomedical Ethics Committee of Peking University (IRB00001052-13074).

Participants in the CLHLS were selected through a stratified random sampling strategy, covering half of the urban cities and rural counties in 22 provinces. Populations in the 22 participating provinces accounted for 85% of the entire population in mainland China (31 provinces). In the CLHLS, gender and age balance was considered in the sampling: approximately equal numbers of male and female nonagenarians (90–99 years), octogenarians (80–89 years) and young-old (65–79 years) were approached in the survey [21]. Households in the participating communities were randomly selected. One elderly participant, if present, from each household was selected for the surveys. On average, a response rate of 98% was reached in the surveys. Full details about the sampling and data collection methods in the CLHLS can be found elsewhere [19,20].

In this study, we used two waves of data, ten years apart: the most recent one (n = 7192) collected in 2014 in comparison with the one (n = 15,638) collected in 2005. These two waves of data were chosen for the following reasons. First, data collected prior to 2005 measured actual use of healthcare services. But since 2005, this has changed to perceived accessibility to healthcare. Second, a long-time interval is required to reveal changes. Third, the two cohorts of samples were almost independent to each other, due to the long time interval, which is important for the application of the Oaxaca–Blinder decomposition method [22].

The questionnaires containing missing values on the outcome and explanatory variables were excluded from data analyses. This resulted in a final sample of 3825 for the 2014 cohort and 11,199 for the 2005 cohort. Only 290 (1.9%) respondents participated in both cohorts of surveys. For simplicity of reporting, we present the results inclusive of the 290 repeated participants. The data analyses excluding the 290 repeated participants generated almost identical results (Supplementary Tables S1 and S2).

### 2.2. Outcome Variable

In this study, we measured perceived accessibility to healthcare. It was measured using a single question: “Could you get adequate medical services when needed?”. Respondents were asked to choose an answer “yes” or “no”. This approach is somehow different from some previous studies, in which the actual use of healthcare services over a given period of time was measured [12]. There is not a golden standard for measuring “access to healthcare”. But it has been widely accepted that this concept can involve multiple dimensions, such as availability, accessibility, accommodation, affordability, and acceptability, depending on how it is measured [23]. The actual use of healthcare services captures “access data” of those in need at the time of the survey. But Cylus and Papanicolas [24] argued that some participants who were not in need at the time may still be able to access healthcare when they need it. This can only be captured by measuring perceived accessibility to healthcare.

### 2.3. Explanatory Variables

The explanatory variables included in this study were categorized in line with the Andersen healthcare utilization model, which classifies determinants of healthcare services into need, predisposing, and enabling factors [25].

The *need* factor indicates whether and what healthcare services are needed by an individual from a medical point of view [25]. The CLHLS captured both subjective and objective indicators of healthcare needs. Respondents were asked to rate their overall health on a five-point Likert scale ranging from “very bad” to “very good”. Meanwhile, they were also asked to confirm whether they had ever been diagnosed by a doctor with hypertension, diabetes, and heart disease, the three most common chronic conditions in China [7].

The predisposing factor determines the inclination of an individual to seek healthcare services [25], which was measured in this study by the demographic characteristics (age, gender, schooling and marital status) and cohabitant living arrangements of the respondents.

The enabling factor activates (or impedes) the realization of healthcare seeking behaviors of those in need [25]. Financial affordability is perhaps the most common enabling factor explored in the literature [26]. In this study, respondents were asked to rate their financial status on a five-point Likert scale (ranging from “very poor” to “very rich”), their affordability for daily expenses (“yes” or “no”), and the out-of-pocket payment ratio for medical care including both outpatient and inpatient care over the last year. We also measured employment status (“retired” vs. “unretired”), the amount of health insurance coverage, residency (urban vs. rural) and geographic location as enabling factors. Over the study period from 2005 to 2014, social health insurance coverage expanded rapidly in China. However, compensations from the insurance programs were often limited due to the small funding pools, prompting people to seek supplementary insurance coverage [14]. There also existed great regional disparities in healthcare services. Rural residents and those who resided in a less-developed region (such as the western and central parts of China) not only had lower income but also enjoyed lower levels of entitlements in welfare including health insurance [27].

### 2.4. Data Analysis

We compared perceived accessibility (%) to healthcare (outcome indicator) between the two cohorts of samples, as well as variations of perceived accessibility with the explanatory variables in 2005 and 2014, respectively, using Chi-square tests.

There existed significant differences in the outcome indicator (*p* < 0.001) and the distributions of all of the explanatory variables (*p* < 0.05) between 2005 and 2014. The contributions of the explanatory variables to the change in the outcome indicator were divided into two components using the Oaxaca–Blinder decomposition method: (1) the endowment portion as a result of distribution differences of the explanatory variables (distributional effect) and (2) the coefficient portion as a result of differential responses of the dependent variable to the explanatory variables (coefficient effect). The Oaxaca–Blinder decomposition method was originally proposed for linear modeling. It has recently been extended to non-linear models [28]. In this study, we adopted a two-step approach to decompose the effects of the explanatory variables using a logistic regression model.

Step one: an aggregate decomposition analysis was performed to identify the total distributional and total coefficient effects after adjustments for the interaction effect between the two.

(1) $${\bar{Y}}_{2014}-{\bar{Y}}_{2005}=\underset{{\hat{\Delta }}_{\beta }^{\mu }}{\underbrace{{\bar{X}}_{2005}({\hat{\beta }}_{2014}-{\hat{\beta }}_{2005})}}+(\underset{{\hat{\Delta }}_{X}^{\mu }}{\underbrace{{\bar{X}}_{2014}-{\bar{X}}_{2005}){\hat{\beta }}_{2005}}}\;+\underset{{\hat{\Delta }}_{I}^{\mu }}{(\underbrace{{\bar{X}}_{2014}-{\bar{X}}_{2005})({\hat{\beta }}_{2014}-{\hat{\beta }}_{2005})}}$$

where ${\hat{\beta }}_{2014}$ and ${\hat{\beta }}_{2005}$ represent the regression coefficients of the explanatory variables in the 2014 and 2005 cohorts, respectively. ${\bar{X}}_{2014}$ and ${\bar{X}}_{2005}$ are the corresponding covariate means of the explanatory variables. ${\hat{\Delta }}_{\beta }^{\mu }$ indicates the total coefficient effect, representing the impacts of the changing regression coefficients. ${\hat{\Delta }}_{X}^{\mu }$ indicates the distributional effect, representing the level of impacts of the explanatory variables estimated at the means. ${\hat{\Delta }}_{I}^{\mu }$ is an interaction between the group differences in the regression coefficients and the distributions of the explanatory variables, as well as the differences in residuals [28,29].

Step two: a detailed decomposition analysis was performed based on the aggregate decomposition to determine the distributional and coefficient effects of each explanatory variable on the change in perceived accessibility to healthcare. ${\hat{\Delta }}_{\beta }^{\mu }$ and ${\hat{\Delta }}_{X}^{\mu }$ were decomposed through the following equations:

(2) $${\hat{\Delta }}_{\beta }^{\mu }=({\hat{\beta }}_{2014,0}-{\hat{\beta }}_{2005,0})+\overset{K}{\underset{K=1}{\sum }}({\hat{\beta }}_{2014,K}-{\hat{\beta }}_{2005,K}){\bar{X}}_{2005,K}$$

and

(3) $${\hat{\Delta }}_{X}^{\mu }=\overset{K}{\underset{K=1}{\sum }}\left({\bar{X}}_{2014,K}-{\bar{X}}_{2005,K}\right){\hat{\beta }}_{2005,K}$$

where ${\hat{\beta }}_{2005,0}$ and ${\hat{\beta }}_{2014,0}$ are the estimated intercepts for 2005 and 2014, respectively. $\;{\bar{X}}_{2005,K}$ and $\;{\bar{X}}_{2014,K}$ represent the means of the *K*th covariate in the corresponding years. Therefore, the contribution of each explanatory variable is a result of the joint effect from a change in the level of the covariate mean and a change in its marginal effect [28,29].

All of the statistical analyses were performed using STATA 14.0. A *p*-value of less than 0.05 was considered statistically significant.

## 3. Results

### 3.1. Changes in Perceived Accessibility to Healthcare

Overall, perceived accessibility to healthcare from the elderly respondents increased from 89.6% in 2005 to 96.7% in 2014, representing a 7.1 percentage point of improvement (*p* < 0.001). Significant variations of the outcome indicator with the explanatory variables were found in the 2005 cohort of samples. Those who were older, female, unmarried, unretired, lived alone, rated poorer health, reported no chronic conditions, felt greater financial difficulties, resided in rural and less-developed regions, and had no insurance coverage perceived lower levels of accessibility to healthcare. Some of the variations, such as those with residency, regional location and chronic conditions became statistically insignificant, while the gender difference became reversed in the 2014 cohort of samples (Table 1).

There were also significant differences in the distributions of the explanatory variables between the two cohorts of samples (Table 1). For example, only 4% of respondents reported no health insurance coverage in 2014, compared with 63.6% in 2005. About 11.6% of respondents rated their financial status as poor or very poor and 17.5% reported being unable to afford daily expenses in 2014, compared with 16.6% and 22.8% in 2005, respectively (Table 1).

### 3.2. Aggregate Decomposition of the Change in Perceived Accessibility to Healthcare

About 82% of the change in perceived accessibility to healthcare was contributed by the coefficient effect, compared with 63% by the distributional effect after adjustments for the negative (−45%) interaction effect (Table 2).

### 3.3. Decomposition of Contributions of Individual Explanatory Variables

**Figure 1:**
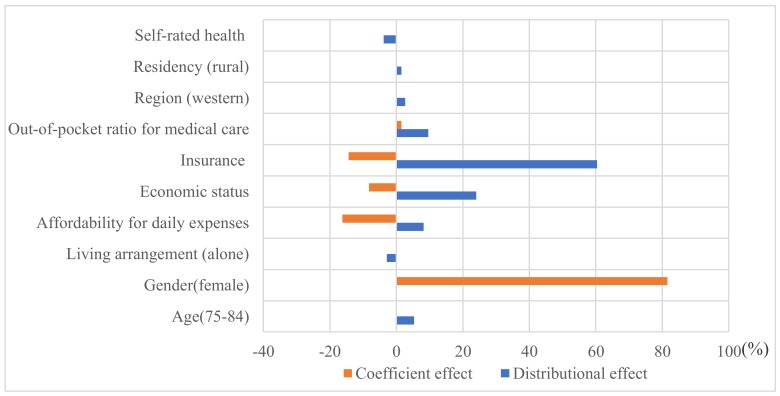
Percentages of distributional and coefficient contributions to the change in perceived accessibility to healthcare between 2005 to 2014.

Distributional changes in age, living arrangements, affordability for daily expenses, self-rated economic status, residency and regional location, insurance coverage, out-of-pocket payment ratio for medical care, and self-rated health made a significant contribution to the change in perceived accessibility (Table 3). The distributional effects on the improvement of perceived accessibility were mainly attributable to increased insurance coverage (60.36%), reduction in self-rated poor economic status (24.05%), reduced out-of-pocket payment ratio for medical care (9.58%), and improved affordability for daily expenses (8.24%) (Figure 1).

The coefficient effects showed that the financial and insurance effects on perceived accessibility became weaker over time, whereas the effects of gender and out-of-pocket payment ratio for medical care became stronger over time (Figure 1).

## 4. Discussion

This study shows that perceived accessibility to healthcare by the elderly in China improved significantly over the period from 2005 to 2014. The change is attributable to both distributional and coefficient effects of a range of explanatory factors.

The proportion of people ≥85 years contributes to the improvement of perceived accessibility to healthcare decreased in the wave of 2014. Consistent with Anthony’s study focusing on the effect of age on preventive healthcare services usage [30], a younger age was also found to be associated with a higher level of inclination to seek healthcare services in the elderly populations. However, the coefficient effect indicates that the overall impact of age on perceived accessibility to healthcare remained unchanged. This means that the further progression of ageing in China could impose a potential risk of declined access to healthcare services.

Gender gaps in perceived accessibility to healthcare are closing, according to the coefficient effect of gender in this study. Elderly women were likely to receive more healthcare services in 2014 than their counterparts were ten years ago. Previous studies have revealed that women tend to have poorer perceived health and higher levels of willingness to seek medical attention than their male counterparts [12,31,32]. But they are also more likely to suppress their healthcare needs when financial hardship hits, and therefore, are more responsive to social support [33,34]. Increased financial subsidies such as the growth of pension and medical insurance coverage may help to relieve their suppressed health needs [15].

The improvement in perceived accessibility to healthcare is attributable to the improved financial capacities of elderly people, according to the distributional effects revealed in this study. Insurance coverage, economic status, out-of-pocket payment ratio for medical care and affordability for daily expenses are the four leading contributors to the distributional effects. Clearly, the rapid socioeconomic development in China over the past two decades has made a significant contribution to the improvement of healthcare services [35,36], in particular, through the social insurance programs [37]. However, it is worth noting that the overall association between wealth and accessibility to healthcare has become weaker as China has become richer, according to the coefficient effects of insurance, economic status and affordability for daily expenses. This finding is echoed by the phenomenon that poorer regions (such as the western part of China and rural areas) demonstrated a positive distributional effect on perceived accessibility of healthcare after adjustments for socioeconomic factors. The weakened effects of financial factors may be explained by the realization of the universal and equitable access to essential medical and public health services [38]. From 2003 to 2011, health insurance coverage in China increased, from 56% for the urban and 21% for the rural populations, to almost 95% for both [39]. By 2014, pension insurance programs covered approximately 80% of the elderly populations [15,40].

However, there is still room for further improvement. Despite a positive distributional effect of (lower) out-of-pocket payment ratio for medical care on perceived accessibility to healthcare, the coefficient effect indicates that the impact of out-of-pocket payment ratio for medical care became stronger over time. Indeed, a significant reduction in out-of-pocket payment ratio occurred over the period from 2000 to 2012 from all of the social health insurance programs [37]. But there existed great disparities in the entitlements across various social health insurance programs [37], leading to increased inequalities in access to healthcare services. For example, the out-of-pocket payment ratio for urban employees was 31.2% in 2013, compared with 49.9% for rural residents according to the China National Household Health Services Survey [41]. The elderly people in China are highly sensitive to out-of-pocket payments in making medical decisions [42].

The distributional effect of living arrangements also deserves increasing policy attention. The elderly who live alone are less likely to access healthcare services than others. The lack of family support creates a serious barrier for them to seek healthcare services [43,44,45]. Although the impact of living arrangements remained unchanged over time according to the coefficient effect found in this study, China is expecting increasing numbers of elderly people living alone. This is a combined result of shrinking family size due to the decades-long family planning policy, scant resources for community and institution care for the elderly, and a growing number of young workers migrating from rural to urban areas [18].

Two limitations should be acknowledged in this study. Firstly, the measurement of healthcare accessibility was based on the perceptions of respondents. This may not accurately reflect their actual use of healthcare services. Further study can consider using actual data to measure health services utilization, such as the number of doctor visits and hospitalizations. Secondly, the explanatory variables had a focus on the perspective of consumers. Variables measuring the perspective of providers, such as the density of health resources and price levels of medical services, were not available. However, these variables can theoretically affect the accessibility to healthcare. Therefore, future research should take them into account, if data are available.

## 5. Conclusions

Overall, perceived accessibility to healthcare from the elderly in China improved from 2005 to 2014. The rapid socioeconomic development contributes significantly to the improvement of healthcare accessibility. However, it is important to note that the impacts of some socioeconomic factors on healthcare accessibility are becoming weaker over time, even though the impact of out-of-pocket payment ratio for medical care seems to be stronger.

Future policy interventions should give priority to equalities across various social health insurance programs. Meanwhile, the significant distributional effect of age on perceived accessibility to healthcare also deserves increasing policy attention. Some healthcare services packages targeted at the older population should be designed for reducing barriers to receiving health care. In addition, the impact of living arrangement also implies that community and institutional care for those living alone needs to be strengthened for improved accessibility to healthcare.

## Figures and Tables

**Table 1:** Perceived accessibility to healthcare in 2005 and 2014 stratified by the explanatory variables.

	2005 (n = 11,199)				2014 (n = 3825)			
	Sample Size N (%)	Access to Healthcare			Sample Size N (%)	Access to Healthcare		
		Yes	%	*p*		Yes	%	*p*
**Age (years)**				<0.001				0.006
65–74	2651 (23.7)	2430	91.66		722 (18.9)	709	98.20	
75–84	2305 (20.6)	2071	89.85		1315 (34.4)	1277	97.11	
≥85	6243 (55.7)	5529	88.56		1788 (46.7)	1713	95.81	
**Gender**				<0.001				0.004
Male	4909 (43.8)	4470	91.06		1816 (47.5)	1753	96.53	
Female	6290 (56.2)	5560	88.39		2009 (52.5)	1946	96.86	
**Living arrangement**				<0.001				<0.001
With family	9500 (84.8)	8613	90.66		3110 (81.3)	3028	97.36	
Alone	1449 (12.9)	1185	81.78		655 (17.1)	613	93.59	
In an institution	250 (2.2)	232	92.80		60 (1.6)	58	96.67	
**Marital status**				<0.001				<0.001
Married	3735 (33.4)	3417	91.49		1601 (41.9)	1568	97.94	
Separated/divorced	263 (2.3)	220	83.65		78 (2.0)	74	94.87	
Widowed	7112 (63.5)	6332	89.03		2115 (55.3)	2033	96.12	
Never married	89 (0.8)	61	68.54		31 (0.8)	24	77.42	
**Years of schooling**				<0.001				0.058
0	6557(58.5)	5753	87.7		2123(55.5)	2040	96.1	
1–5	2761(24.7)	2494	90.3		962(25.2)	938	97.5	
≥6	1881(16.8)	1783	94.8		740(19.3)	721	97.4	
**Employment status**				<0.001				0.007
Unretired	8656 (77.3)	7556	87.19		3110 (81.3)	2996	96.33	
Retired	2543 (22.7)	2474	97.29		715 (18.7)	703	98.32	
**Affordability for daily expenses**				<0.001				<0.001
Yes	8647 (77.2)	8221	95.07		3157 (82.5)	3114	98.64	
No	2552 (22.8)	1809	70.89		668 (17.5)	585	87.57	
**Economic status**				<0.001				<0.001
Very rich	144 (1.3)	140	97.22		61 (1.6)	61	100.00	
Rich	1695 (15.1)	1670	98.53		578 (15.1)	574	99.31	
Fair	7494 (66.9)	7041	93.96		2741 (71.7)	2697	98.39	
Poor	1570 (14.0)	1069	68.09		371 (9.7)	318	85.71	
Very poor	296 (2.6)	110	37.16		74 (1.9)	49	66.22	
**Insurance**				<0.001				0.007
No	7124 (63.6)	6141	86.20		153 (4.0)	143	93.46	
One	2156 (19.3)	2004	92.95		2270 (59.3)	2187	96.34	
Two or above	1919 (17.1)	1885	98.23		1402 (36.7)	1369	97.65	
**Out-of-pocket payment ratio for medical care**				<0.001				0.038
<40%	1217 (10.9)	1174	96.5		1977 (51.7)	1920	97.1	
40–80%	604 (5.4)	572	94.7		777 (20.3)	748	96.3	
>80%	9378 (83.7)	8284	88.3		1071 (28.0)	1031	96.3	
**Region**				<0.001				0.875
Eastern	6574 (58.7)	5973	90.86		2109 (55.1)	2039	96.68	
Central	2773 (24.8)	2432	87.70		1276 (33.4)	1236	96.87	
Western	1852 (16.5)	1625	87.74		440 (11.5)	424	96.36	
**Residency**				<0.001				0.147
Urban	4985 (44.5)	4667	93.62		1791 (46.8)	1740	97.15	
Rural	6214 (55.5)	5363	86.31		2034 (53.2)	1959	96.31	
**Hypertension**				0.083				0.569
Yes	2266 (20.2)	2052	90.56		1428 (37.3)	1384	96.92	
No	8933 (79.8)	7978	89.31		2397 (62.7)	2315	96.58	
**Diabetes**				0.006				0.240
Yes	349 (3.1)	328	93.98		249 (6.5)	244	97.99	
No	10850 (96.9)	9702	89.42		3576 (93.5)	3455	96.62	
**Heart disease**				0.077				0.790
Yes	1184 (10.6)	1078	91.05		605 (15.8)	584	96.53	
No	10,015 (89.4)	8952	89.39		3220 (84.2)	3115	96.74	
**Self-rated health**				<0.001				<0.001
Very good	1118 (10.0)	1073	95.97		306 (8.0)	304	99.35	
Good	4171 (37.2)	3887	93.19		1278 (33.4)	1260	98.59	
Fair	3893 (34.8)	3481	89.42		1546 (40.4)	1491	96.44	
Bad	1812 (16.2)	1444	79.69		628 (16.4)	589	93.79	
Very bad	205 (1.8)	145	70.73		67 (1.8)	55	82.09	

**Table 2:** Aggregate decomposition of the change in perceived accessibility to healthcare.

	Coefficient	Percentage Contribution (%)
Accessibility in 2014	0.9670 **	
Accessibility in 2005	0.8956 **	
Change in accessibility	0.0714 **	
**Overall contribution to the change**		
Distributional effect	0.0449 **	63
Coefficient effect	0.0585 **	82
Interaction	−0.0319 **	−45

Notes: ** *p* < 0.001.

**Table 3:** Distributional and coefficient effects of explanatory variables on the change in perceived accessibility to healthcare.

Explanatory Variable	Distributional Effect		Coefficient Effect		Interaction Effect	
	Coefficient	%	Coefficient	%	Coefficient	%
**Age (years)** (Ref. = “≥85”)						
65–74	−0.0017	−3.79	0.0046	7.86	−0.0017	5.33
75–84	0.0024 *	5.35	0.0025	4.27	0.0033	−10.34
**Gender** (Ref. = Male)						
Female	0.0006	1.34	0.0477 *	81.54	−0.0020	6.27
**Living arrangement** (Ref. = With family)						
Alone	−0.0013 *	−2.90	−0.0014	−2.39	−0.0009	2.82
In an institution	−0.0003	−0.67	−0.0004	−0.68	0.0002	−0.63
**Marital status** (Ref. = Married)						
Separated/Divorce	0.0001	0.22	0.0001	0.17	−0.0001	0.31
Widowed	−0.0008	−1.78	−0.0162	−27.69	0.0040	−12.54
Never married	−0.0001	−0.22	0.0001	0.17	0.0001	−0.31
**Years of schooling** (Ref. = 0)						
1–5	−0.0001	−0.22	0.0061	10.43	0.0003	−0.94
≥6	−0.0002	−0.45	0.0002	0.34	0.0001	−0.31
**Employment status** (Ref. = Unretired)						
Retired	−0.0002	−0.45	−0.0071	−12.14	0.0005	−1.57
**Affordability for daily expenses** (Ref. = No)						
Yes	0.0037 **	8.24	−0.0095 *	−16.24	0.0012	−3.76
**Economic status** (Ref. = Very poor)						
Poor	0.0033	7.35	−0.0009	−1.54	0.0005	−1.57
Fair	0.0108 *	24.05	−0.0006	−1.03	−0.0001	0.31
Rich	0.0006	1.34	−0.0018	−3.08	−0.0001	0.31
Very rich	0.0025	5.57	−0.0048 **	−8.21	0.0034	−10.66
**Insurance** (Ref. = No)						
One	0.0094 *	20.94	−0.0024	−4.10	−0.0099	31.03
Two or more	0.0177 **	39.42	−0.0084 *	−14.36	−0.0186 *	58.31
**Out-of-pocket ratio for medical care** (Ref. = “≥80%”)						
<40%	0.0043 *	9.58	0.0009 *	1.54	−0.0035	10.97
40–79%	0.0020	4.45	0.0004	0.68	−0.0048	15.05
**Region** (Ref. = Eastern)						
Central	−0.0102	−22.72	0.0047	8.03	0.0033	−10.34
Western	0.0012 *	2.67	0.0022	3.76	−0.0013	4.08
**Residency** (Ref. = Urban)						
Rural	0.0007 *	1.56	0.0312	53.33	−0.0007	2.19
**Hypertension** (Ref.=Yes)						
No	0.0010	2.23	−0.0031	−5.30	0.0005	−1.57
**Diabetes** (Ref. = Yes)						
No	0.0006	1.34	−0.0155	−26.50	0.0005	−1.57
**Heart disease** (Ref. = Yes)						
No	0.0004	0.89	0.0293	50.09	−0.0015	4.70
**Self-rated health** (Ref. = Very bad)						
Bad	0.0001	0.22	0.0018	3.08	0.0001	−0.31
Fair	0.0031 *	6.90	0.0029	4.96	0.0009	−2.82
Good	−0.0027 *	−6.01	0.0093	15.90	−0.0018	5.64
Very good	−0.0021 *	−4.68	0.0044	7.52	−0.0020	6.27

Note: Figures in bold indicate coefficients with statistical significance * *p* < 0.05; ** *p* < 0.001.

## Supplementary Materials

The following are available online at https://www.mdpi.com/1660-4601/16/20/3824/s1, Table S1: Aggregate decomposition of the change in perceived accessibility to healthcare (excluding the 290 repeated respondents), Table S2: Distributional and coefficient effects of explanatory variables on the change in perceived accessibility to healthcare (excluding the 290 repeated respondents).
