# Supplementary material for: Changes in Perceived Accessibility to Healthcare from the Elderly between 2005 and 2014 in China: An Oaxaca–Blinder Decomposition Analysis
Source: Int J Environ Res Public Health. 2019 Oct 10;16(20):3824. doi: 10.3390/ijerph16203824 (PMC6843178; doi:10.3390/ijerph16203824)
Supplement: Supplementary file 1 [file ijerph-16-03824-s001.pdf]

Supplementary Tables

Table S1. Aggregate decomposition of the change in perceived accessibility to healthcare (excluding the 290 repeated respondents).

	Coefficient	Percentage contribution (%)
Accessibility in 2014	0.9668**	
Accessibility in 2005	0.8932**	
Change in accessibility	0.0735**	
**Overall contribution to the change**		
Distributional effect	0.0471**	64
Coefficient effect	0.0602**	82
Interaction	-0.0339**	-46

Notes: ** *p < 0.001*

Table S2. Distributional and coefficient effects of explanatory variables on the change in perceived accessibility to healthcare (excluding the 290 repeated respondents)

Explanatory variable	Distributional effect		Coefficient effect		Interaction effect	
	Coefficient	%	Coefficient	%	Coefficient	%
**Age (years)** (Ref.="≥85")						
65-74	-0.0001	-0.21	0.0042	6.98	-0.0001	0.29
75-84	**0.0013***	2.76	0.0022	3.65	0.0015	-4.42
**Gender** (Ref.=Male)						
Female	0.0090	19.11	**0.0323***	53.65	-0.0020	5.90
**Living arrangement** (Ref.=With family)						
Alone	**-0.0012***	-2.55	-0.0004	-0.66	-0.0003	0.88
In an institution	-0.0003	-0.64	-0.0004	-0.66	0.0003	-0.88
**Marital status** (Ref.=Married)						
Separated/Divorce	0.0001	0.21	0.0002	0.33	-0.0001	0.29
Widowed	-0.0011	-2.34	-0.0158	-26.25	0.0045	-13.27
Never married	0.0001	0.21	-0.0002	-0.33	0.0001	-0.29
**Years of schooling (**Ref.=**0)**						
1-5	0.0001	0.21	0.0076	12.62	-0.0003	0.88
≥6	-0.0003	-0.64	0.0014	2.33	0.0006	-1.77
**Employment status** (Ref.=Unretired)						
Retired	-0.0001	-0.21	-0.0228	-37.87	0.0008	-2.36
**Affordability for daily expenses** (Ref.=No)						
Yes	**0.0049****	10.40	**-0.0116***	-19.27	0.0020	-5.90
**Economic status** (Ref.=Very poor)						
Poor	0.0039	8.28	-0.0010	-1.66	-0.0007	2.06
Fair	**0.0113***	23.99	-0.0081	-13.46	0.0014	-4.13
Rich	0.0025	5.31	-0.0016	-2.66	-0.0005	1.47
Very rich	0.0024	5.10	**-0.0040****	-6.64	0.0032	-9.44
**Insurance** (Ref.=No)						
One	**0.0104***	22.08	-0.0037	-6.15	-0.0164	48.38
Two or more	**0.0161****	34.18	**-0.0085***	-14.12	**-0.0187***	55.16
**Out-of-pocket ratio for medical care** (Ref.= "≥80%")						

<40%	**0.0056**[*]	11.89	**0.0015**[*]	2.49	-0.0035	10.32
40%-79%	0.0011	2.34	0.0004	.66	-0.0067	19.76
**Region** (Ref.=Eastern)						
Central	-0.0011	-2.34	0.0069	11.46	0.0050	-14.75
Western	**0.0021**[*]	4.46	0.0016	2.66	-0.0017	5.01
**Residency** (Ref.=Urban)						
Rural	**0.0008**[*]	1.70	0.0270	44.85	0.0002	-.59
**Hypertension** (Ref.=Yes)						
No	0.0012	2.55	0.0109	18.11	-0.0020	5.90
**Diabetes** (Ref.=Yes)						
No	0.0005	1.06	-0.0301	-50.00	0.0033	-9.73
**Heart disease** (Ref.=Yes)						
No	0.0004	0.85	0.0313	51.99	-0.0017	5.01
**Self-rated health** (Ref.=Very bad)						
Bad	-0.0003	-0.64	0.0006	1.00	0.0001	-0.29
Fair	**0.0031**[*]	6.58	0.0034	5.65	-0.0011	3.24
Good	**-0.0018**[*]	-3.82	0.0012	1.99	-0.0002	0.59
Very good	**-0.0018**[*]	-3.82	0.0027	4.49	-0.0011	3.24

Note: Figures in bold indicate coefficients with statistical significance * $p < 0.05$; ** $p < 0.001$
